# Immunogenicity and Protective Efficacy against Murine Tuberculosis of a Prime-Boost Regimen with BCG and a DNA Vaccine Expressing ESAT-6 and Ag85A Fusion Protein

**DOI:** 10.1155/2011/617892

**Published:** 2011-02-27

**Authors:** Jia Lu, Chun Wang, Zhiguang Zhou, Ying Zhang, Tingting Cao, Chunwei Shi, Zhenhua Chen, Lingxia Chen, Changxue Cai, Xionglin Fan

**Affiliations:** ^1^Laboratory of Biosafety, Department of Pathogen Biology, Tongji Medical College, Huazhong University of Science & Technology, no.13, Hangkong Road, Wuhan 430030, China; ^2^Department of Molecular Microbiology and Immunology, Bloomberg School of Public Health, Johns Hopkins University, Baltimore, MD 21205, USA

## Abstract

Heterologous prime-boost regimens utilizing BCG as a prime vaccine probably represent the best hope for the development of novel tuberculosis (TB) vaccines. In this study, we examined the immunogenicity and protective efficacy of DNA vaccine (pcD685A) expressing the fusion protein of Ag85A and ESAT-6 (r685A) and its booster effects in BCG-immunized mice. The recombinant r685A fusion protein stimulated higher level of antigen-specific IFN-*γ* release in tuberculin skin test- (TST-) positive healthy household contacts of active pulmonary TB patients than that in TST-negative population. Vaccination of C57BL/6 mice with pcD685A resulted in significant protection against challenge with virulent *Mycobacterium tuberculosis* H37Rv when compared with the control group. Most importantly, pcD685A could act as a BCG booster and amplify Th1-type cell-mediated immunity in the lung of BCG-vaccinated mice as shown the increased expression of IFN-*γ*. The most significant reduction in bacterial load of both spleen and lung was obtained in mice vaccinated with BCG prime and pcD685A DNA booster when compared with BCG or pcD685A alone. Thus, our study indicates that pcD685A may be an efficient booster vaccine against TB with a strong ability to enhance prior BCG immunity.

## 1. Introduction

Tuberculosis (TB), caused by *Mycobacterium tuberculosis*, is a leading infectious killer worldwide [1]. The emergence and spread of multidrug-resistant TB (MDR-TB) and extensively drug-resistant TB (XDR-TB) and coinfection with TB/HIV pose serious challenges to effective TB control [2]. *M. bovis* BCG is currently the only available vaccine against TB, with over 120 million doses administered annually [3]. BCG immunization provides protection against severe forms of TB in children, including tuberculous meningitis and miliary TB [4]. However, BCG induced-protection lasts less than 15 years because antituberculous protective immunity wanes gradually after the initial immunization [5]. Consequently, developing new, more effective vaccines and immunization strategies aimed to boost waning BCG-induced protective responses, is urgently needed.

DNA vaccines can stimulate both humoral and cell-mediated immunity in different animal models of TB and is thought to be a promising strategy in the development of new vaccines against TB [6]. DNA vaccine candidates expressing several antigens of *M. tuberculosis* have been shown to provide protective immune responses against TB [6] and to boost BCG efficacy using prime/boost strategies [7]. In our previous study, we constructed two DNA vaccine candidates separately encoding antigen Ag85A and ESAT-6 from *M. tuberculosis,* and both DNA vaccines could induce strong humoral and cell-mediated immunity in vaccinated mice, which resulted in some degree of protection in mice challenged with virulent *M. tuberculosis* [8]. DNA vaccine expressing ESAT-6 protein could enhance the protective efficacy of BCG vaccination in mice vaccinated with a combination strategy of BCG and DNA vaccine [9]. In the present study, we evaluated the immune responses generated against DNA vaccine expressing the fusion protein of ESAT-6 and Ag85A (r685A) and the immunogenicity of r685A fusion protein in tuberculin skin test- (TST-) positive healthy populations. In addition, we evaluated the use of a BCG prime plus DNA vaccine in a prime/boost strategy to induce protection against virulent *M. tuberculosis* challenge in mice.

## 2. Materials and Methods

### 2.1. Bacterial Strain and Culture Media

*Escherichia coli* DH5*α* and BL21 (DE3) strains were used for cloning and overexpression, respectively. Both bacteria were cultured in Luria-Bertani (LB) medium with or without agar. When required, ampicillin was added to a final concentration of 100 *μ*g/mL. *M. tuberculosis* H37Rv and *M. bovis* BCG China were cultivated in Middlebrook 7H9 medium or enumerated on 7H11 agar (BD, Sparks, USA), supplemented with 10% ADC, 0.5% glycerol, and 0.05% Tween 80.

### 2.2. Construction of Recombinant Plasmids

Genes coding ESAT-6 (*esxA, Rv3875*) and Ag85A (*fbpA, Rv3804c*) were amplified, respectively, by PCR with primers (listed in Table 1) and the genomic DNA of *M. tuberculosis* H37Rv as the template. The gene encoding the fusion protein of ESAT-6 and Ag85A was generated by a second PCR according to the gene splicing with the overlap extension (GeneSOEing) method [10]. The PCR products were first digested with *Bam*HI and *Eco*RI and then cloned into the corresponding sites of prokaryotic expression vector pProEXHTb (Invitrogen, Carlsbad, CA, USA) and eukaryotic expression vector pcDNA3.1(+), resulting in recombinant plasmids named pPro685A and pcD685A, respectively. The correctness of recombinant plasmids was confirmed by DNA sequencing and enzyme digestion. Plasmids pcDNA3.1(+) and pcD685A for DNA immunization were transformed into competent *Escherichia coli* DH5*α*, and endotoxin-free plasmid DNA was purified using the Qiagen Plasmid Giga kit (Qiagen, Valencia, CA) according to the manufacturer's instructions.

### 2.3. Overexpression of r685A Protein in E. coli

*E. coli* BL21 (DE3) strain harboring the plasmid pPro685A was cultured overnight. Overnight cultures were inoculated into fresh LB medium (1 : 100) containing ampicillin and incubated at 37°C with shaking, until OD_600_ nm reached 0.6. The expression of the fusion protein r685A was induced with isopropyl thio-*β*-D-galactoside (IPTG) at a final concentration of 0.1 mM for 0, 2, 4, or 6 hours. Bacterial pellets were collected by centrifugation, prepared in sample buffer, and subjected to SDS-PAGE analysis. Proteins were stained with a Coomassie blue dye. 

Purification of r685A protein was performed using the Ni-NTA Purification System according to the manual instructions (Invitrogen, USA). The purified r685A protein was lyophilized and diluted in normal saline, using pyrogen-free reagents, and tested to exclude endotoxin contamination. The protein concentration was determined by using the Enhanced BCA Protein Assay Kit (Beyotime Institute of Biotechnology, Haimen, China) and stored at −20°C. 

To verify the specificity of purified r685A fusion protein, the protein samples were first separated by SDS-PAGE and then transferred onto nitrocellulose membranes and probed with a rabbit polyclonal antibody to ESAT-6 (Abcam 45073, Cambridge Science Park, Cambridge, UK) and a chicken polyclonal antibody to Ag85A (Abcam 36731, Cambridge Science Park, Cambridge, UK) as the primary antibody. Alkaline phosphatase-conjugated goat antirabbit IgG antibody and rabbit antichicken IgG antibody (Santa Cruz Biotech., Santa Cruz, CA, USA) were as the secondary antibody, respectively.

### 2.4. Overexpression of r685A Protein In Vitro

In vitro overexpression of r685A fusion protein was verified by Western blot analysis of transfected HeLa cells. Cells were cultured in wells of a 6-well plate and transfected with 1 *μ*g of pcD685A or empty vector (pcDNA3.1). G418-resistant colonies (250 mg/L) were selected and harvested for the Western blotting analysis with mouse polyclonal antibody to r685A.

### 2.5. Immunogenicity of r685A Protein

The study protocol was approved by the Ethics Committee of Tongji Medical College. Written informed consents were obtained from all subjects involved in this study. Seventeen household contacts with recent sputum-positive TB patients (mean age: 37  ±  19 years; male/female ratio: 7/10) were enrolled. Active TB was excluded for all household contacts based on radiologic and clinical examinations, sputum microscopy, and bacteriologic culture. After heparinized whole blood sample from each participants was collected, TST and whole blood IFN-*γ* assay (WBIA) based on the r685A protein were performed as previously described, respectively [11]. Reactions of <5 mm and ≥5 mm were considered TST negative and positive, respectively. Whole blood from each donor (1 mL) was seeded in 24-well plates and incubated with 20 *μ*L r685A protein at final concentrations of 0 or 10 *μ*g/mL for 24 h at 37°C. After stimulation, 200 *μ*L of plasma was then taken from each well and stored at −20°C until use. The concentrations of IFN-*γ* in collected samples were determined in duplicate, using a commercial enzyme-linked immunosorbent assay kit according to the manufacturer's instructions (Dakewei Biotech, Shenzhen, China).

### 2.6. Animal Immunization

Specific pathogen-free, 6- to 8-week-old, female C57BL/6 mice (Vital River Lab Animal, Beijing, China) were bred in cages on the animal feeding cabinet (VentiRack, Chester, CA, USA) in a biosafety level 3 laboratory. Mice received free access to food and water throughout the study. The research protocol was reviewed and approved by Tongji Medical School Committees on Biosaftey and Animal Care and Use Committee of China.

Mice were randomly divided into (12 mice in each group): nonvaccinated control, vector control, pcD685A, BCG, BCG prime plus vector booster, and BCG prime plus pcD685A booster. Mice were injected with 30 *μ*L of 0.25% Bupivacaine in the quadriceps muscle of each hind leg three days before DNA immunization. Plasmid DNA (50 *μ*g) was injected intramuscularly in the same area, and immunization was repeated thrice with 2-week intervals. The latter two groups were firstly immunized with 10^6^ CFU BCG once subcutaneously on the first day, then boosted with the plasmids following the DNA vaccination process. Two weeks after the completion of gene immunization, five mice of each group were used for immunological analysis, while the others were challenged with virulent *M. tuberculosis* H37Rv.

### 2.7. Antibody Response

Sera were collected from each mouse two weeks after immunization. Antigen-specific antibody responses were measured in an ELISA using microtiter plates, precoated overnight at 4°C with 100 *μ*L r685A protein (5 *μ*g/mL) in carbonate/bicarbonate buffer (pH 9.6). After blocking with 1% BSA in PBS, serum samples were diluted to appropriate concentrations and were incubated for 2 h at 37°C. After washing, the plates were incubated for 2 h at 37°C with HRP-conjugated goat antimouse IgG antibody. Orthophenylenediamine (OPD) was used for color development as an indicator. Sera from naive mice were used as negative controls. Data are presented as mean of optical density value at 490 nm per group.

### 2.8. qRT-PCR Analysis of IFN-*γ* and IL-10 Expression in Lungs of Vaccinated Mice

About 100 mg of lung tissue was crushed with a syringe plunger and the DNA-free RNA samples were extracted with TRIzol reagent (Invitrogen). For qRT-PCR, 2 *μ*g of RNA was reverse transcribed with M-MLV Reverse Transcriptase (Promega, USA) to obtain cDNA samples. For the real-time reaction, each primer (250 nM) and 7.5 *μ*L of template reaction (1 : 20 dilution) in 25 *μ*L volume with SYBR Green kit (Roche, USA) were used. Triplicate samples were run on an Applied Rotor-Gene 3000 Real-Time system (Gene, USA). Tested cDNAs were normalized to the endogenous RNA levels of the internal control GAPDH. Gene expression was determined using the relative quantification ΔΔCT =   (CT_Test_  −  CT_GAPDH_)_sample_   −  (CT_Test_  −  CT_GAPDH_)_Control_. CT is the fractional cycle number that reaches a fixed threshold, CT_Test_ is the test of each vaccinated group, and CT_Control_ is the reference control from the control group. ΔΔCT is the difference between gene expression in each vaccinated group and the control group. The increase fold was calculated using 2^−ΔΔCT^. Primer sequences and cycle parameters used for qRT-PCR are listed in Supplementary Table 1 (see Supplementary Table 1 in Supplementary Material available online at doi:10:1155/2011/61789g). 

### 2.9. M. tuberculosis Challenge

C57BL/6 mice were infected intravenously through the lateral tail vein at the dose of 10^6^ CFU with exponentially growing *M. tuberculosis* suspended in 100 *μ*L PBS. Four weeks later, seven mice per group were killed, and bacterial burden was determined by plating serial dilutions of lung and spleen homogenates onto Middlebrook 7H11 agar plates supplemented with 10% (v/v) ADC enrichment and 0.5% (v/v) glycerol, containing 2-thiophenecarboxylic acid hydrazide (10 *μ*g/mL), which inhibits residual BCG but not *M. tuberculosis*. Mouse protocols were approved by the Biosafety Committee of Tongji Medical College.

Right lung lobes from different vaccine groups were fixed in 10% PBS-buffered formalin, embedded in paraffin, sectioned and stained with hematoxylin and eosin (HE) followed with analyzing by a pathologist with no prior knowledge for the treatment group and recording the results under a light microscope.

### 2.10. Statistical Analysis

Student's *t* test was used to compare the mean organ burdens of each group of mice, and a *P* value was less than  .05 was considered statistically significant.

## 3. Results

### 3.1. Construction and Overexpression of Recombinant r685A Protein in E. coli

**Figure 1:**
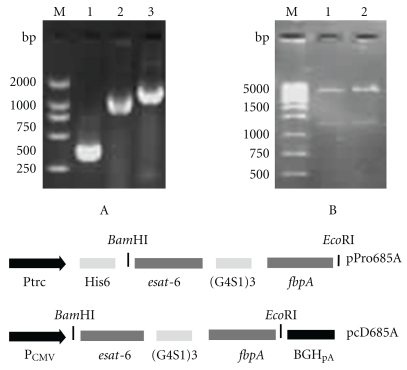
Construction of recombinant plasmids. Genes encoding ESAT-6, Ag85A, the fusion protein of ESAT-6, and Ag85A with a 45 bp linker, were cloned from *M. tuberculosis* H37Rv genomic DNA, respectively (A). The PCR products of the fusion genes were inserted into the *Bam*HI and *Eco*RI sites of pProExHTb or pcDNA3.1, resulting in the recombinant plasmids pPro685A and pcD685A, respectively. The recombinant plasmids were identified by enzyme digestion (B). Lane M: DNA molecular marker; lane A1: PCR product of *esat-6*; lane A2: PCR product of *fbpA*; lane A3: PCR product of *esat-6-fbpA*; lane B1: products of pPro685A digested with enzymes; lane B2: products of pcD685A digested with enzymes.

The genes of ESAT-6 and Ag85A were first amplified by PCR and *M. tuberculosis* H37Rv genomic DNA as the template (Figure 1). The fusion gene of *esat-6* and *fbpA* was then amplified using a mixture of PCR products of *esat-6* and *fbpA* as template with the upstream primer of ESAT-6 and the downstream primer of Ag85A (Figure 1). The purified PCR productions were digested with *Bam*HI and *Eco*RI and separately cloned into the plasmid pProEXHTb and pcDNA3.1(+) predigested with the same restriction enzymes, in order to construct prokaryotic expression plasmid pPro685A and eukaryotic expression plasmid pcD685A (Figure 1). DNA sequencing and enzyme digestion confirmed the successful constructions (Figure 1).

**Figure 2:**
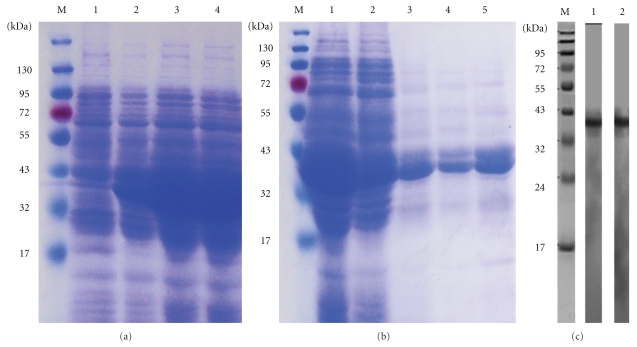
Expression, purification, and identification of recombinant fusion protein. *E. coli* BL21 (DE3) harboring pPro685A was cultured with IPTG. The expression (a) and the purification procession of r685A fusion protein (b) were confirmed by SDS-PAGE and Western blotting (c). Lane M: protein molecular size marker (kDa); lane a1: *E. coli* strain without IPTG; lane a2: *E. coli* strain 2 h after IPTG induction; lane a3: *E. coli* strain 4 h after IPTG induction; lane a4: *E. coli* strain 6 h after IPTG induction; lane b1: cell lysis; lane b2: fraction from Ni-NTA column after wash with denaturing binding buffer; lanes b3 and b4: fraction after wash with wash buffer; lane b5: r685A protein eluted with native elution buffer; lane c1: anti-ESAT-6 antibody; lane c2: anti-Ag85A antibody.

*E. coli* BL21 (DE3) strain containing plasmid pPro685A was induced with IPTG, and recombinant fusion protein r685A was identified by 12% SDS-PAGE with a molecular mass of about 38 kDa as expected (Figure 2). r685A protein was expressed in *E. coli* in the inclusion body, and purification was performed using the Ni-NTA purification system. The process of purification was monitored by SDS-PAGE (Figure 2). The specificity of the purified r685A protein was verified by Western blotting with anti-ESAT-6 and anti-Ag85A antibodies, respectively, (Figure 2).

### 3.2. Expression of pcD685A DNA Construct

**Figure 3:**
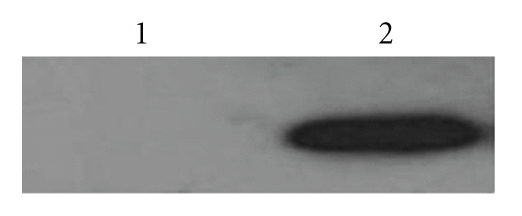
Western blot analysis of cell lysates from Hela cells transfected with the pcD685A construct (lane 2) or empty vector (lane 1). The primary antibody used was mouse antisera after pcD685A DNA vaccination.

After plasmid pcD685A was transfected into HeLa cells by electroporation, cell lines of stable resistance to G418 were obtained by G418 selection. The expression of protein r685A was confirmed by Western blotting (Figure 3). HeLa cells transfected with vector pcDNA3.1(+) were also detected as control and showed the negative result as expected (Figure 3).

### 3.3. Immunogenicity of r685A Protein

**Figure 4:**
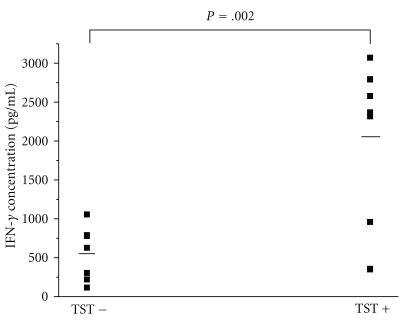
Immunogenicity of r685A fusion protein in healthy population. A total of 17 household contacts with active TB patients were tested by TST and r685A-WBIA, respectively. Each square represents the IFN-*γ* concentration in a sample, and median values for TST− (*n*  =  7) and TST+ (*n*  =  10) groups are indicated by horizontal lines.

Whole blood samples were collected from 17 household contacts with active TB patients and subjected to r685A-WBIA (Figure 4). The levels of IFN-*γ* in all samples without antigen stimuli were below 30 pg/mL (10.6  ±  5.9 pg/mL) but significantly increased to 1401.1  ±  1084.3 pg/mL after r685A protein stimulation. Analysis of the distribution of IFN-*γ* levels in TST-positive and TST-negative samples showed a significant correlation between TST positivity and IFN-*γ* level (Figure 4). The mean IFN-*γ* levels in samples from TST-positive group detected by r685A-WBIA were significantly higher than those for TST-negative group (*P* < .05).

### 3.4. Antibody Response

**Figure 5:**
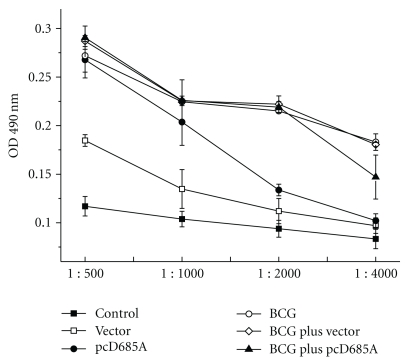
r685A-specific IgG antibody-induced in immunized mice. Mice were immunized and bled at day 14 following the last immunization; serum antibody levels were assessed by ELISA. Sera from five animals in each group were evaluated individually at the dilutions indicated. Results shown are the mean and standard error.

Sera IgG antibodies against r685A protein were determined by ELISA. As shown in Figure 5, strong IgG response to r685A protein was elicited in BCG, pcD685A, BCG plus vector, and BCG plus pcD685A-treated mice groups. As expected, empty DNA vector and control groups stimulated little or very weak IgG response.

### 3.5. Expression of IFN-*γ* and IL-10 in Lungs

**Figure 6:**
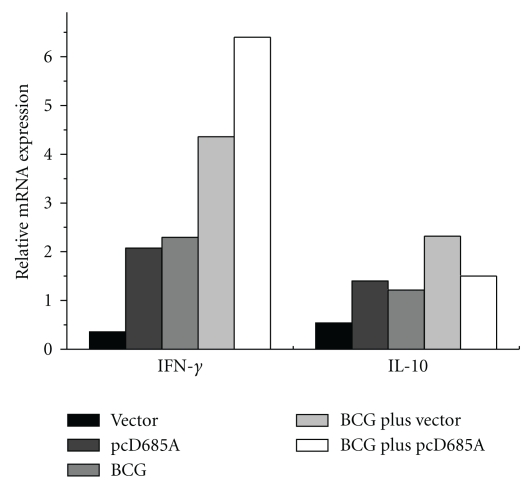
Differential expression of IFN-*γ* and IL-10 in the lungs of vaccinated mice (*n*  =  3). Two weeks after DNA vaccination, RNA extracted from lung of each mouse in different groups was used for the detection of cytokines mRNA concentrations by qRT-PCR analysis. The levels of the cytokine mRNAs for each group were normalized based on the levels of GAPDH. Cytokine mRNA levels were expressed as values relative to the control group.

To determine cell-mediated immune responses in the lung, lung tissues from immunized mice were prepared 2 weeks after the last DNA vaccination. The expression levels of IFN-*γ* and IL-10 in lungs of vaccinated mice were determined by qRT-PCR. As shown in Figure 6, IFN-*γ* response increased in all groups except the vector control group. Mice vaccinated with BCG plus pcD685A induced the highest levels of IFN-*γ* responses in the lungs. Combination with BCG increased significantly the expression of IFN-*γ* when compared with BCG or pcD685A alone. In addition, BCG plus vector group showed the most significant IL10 response in the lung, and high expression of IFN-*γ* was also produced. The expression of both IFN-*γ* and IL-10 decreased in vector group when compared with control mice.

### 3.6. BCG Prime Plus pcD685A Booster Produced Better Protective Effect against Virulent Challenge of M. tuberculosis in Mice than BCG Alone

**Figure 7:**
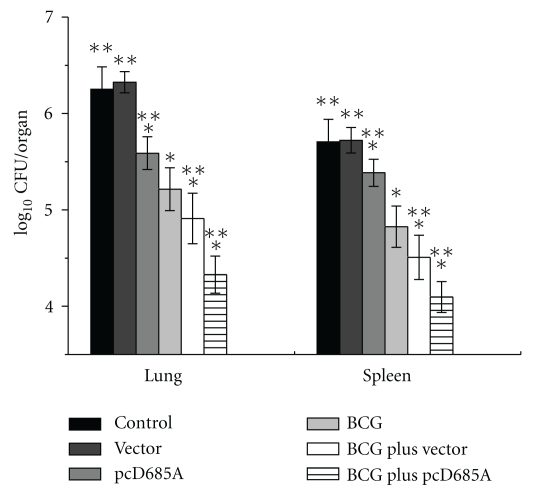
Bacterial load per lung and spleen in C57BL/6 mice at 4 weeks after challenge (*n*  =  7). Vaccinated C57BL/6 mice (*n*  =  7) were challenged i.v. with 10^6^ CFU virulent *M. tuberculosis* H37Rv strain. Four weeks after challenge, lungs and spleens were harvested aseptically and numbers of bacterial CFU per organ were enumerated. Results are shown as the mean (±SEM) log_10_ CFU/organ. *P < .05 versus control; **P < .05 versus BCG. This experiment was repeated twice with similar results.

In order to determine the protection effect, mice were challenged with *M. tuberculosis* H37Rv. The results of bacterial load in different organs were shown in Figure 7. Most importantly, mice vaccinated with BCG plus pcD685A produced the strongest protective effect and significantly inhibited the growth of *M. tuberculosis* in the lung (−1.922 log) and spleen (−1.609 log) when compared with BCG or pcD685A alone (*P* < .05). In addition, pcD685A also evoked a significant protection (−0.663 log) in the lung. Highest bacterial load was seen in the lung and spleen in both control and empty vector groups.

### 3.7. Decreased Lung Pathology in Mice Vaccinated with BCG Plus pcD685A Compared with BCG

**Figure 8:**
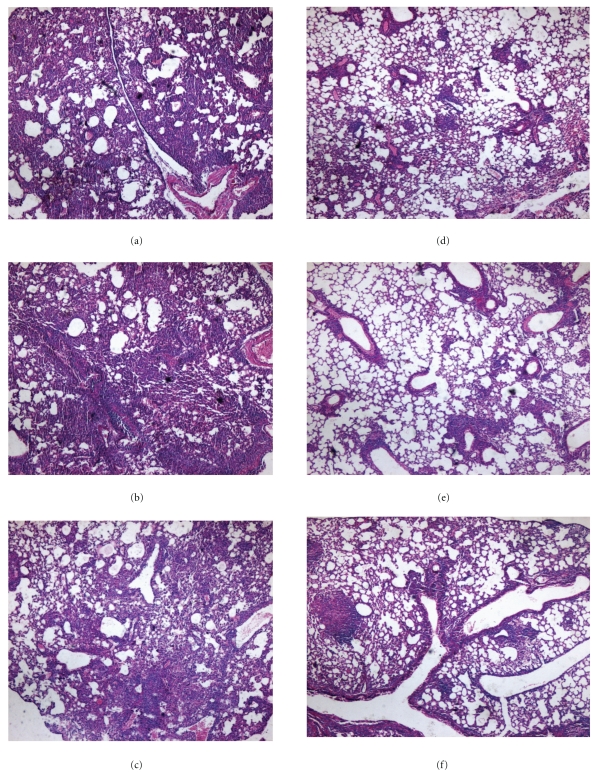
Representative lung pathology of C57BL/6 mice after challenge. Vaccinated C57BL/6 mice were challenged i.v with 10^6^ CFU virulent *Mycobacterium tuberculosis* H37Rv strain. Four weeks after infection, lung tissue sections from different vaccine groups were prepared for HE staining (amplification 10  ×  4), (a) control; (b) vector control; (c) pcD685A; (d) BCG; (e) BCG plus vector; (f) BCG plus pcD685A.

Consistent with varying bacterial loads in the lung, HE-stained sections from different groups showed clear differences (Figure 8). The lung sections from both control and vector control mice showed severe interstitial pneumonia and intense inflammation throughout the lung with the appearance of early granuloma formation (Figures 8(a) and 8(b)). Slight damage in alveolar tissues with aggregated, relatively large number of lymphocytes was also observed in the lung from single pcD685A-vaccinated group (Figure 8(c)). BCG- and BCG plus vector-vaccinated mice showed slight interstitial pneumonia (Figures 8(d) and 8(e)). Alveolar tissue from mice vaccination with BCG plus pcD685A combination appeared to be intact with very limited lung inflammation (Figure 8(f)).

## 4. Discussion

Developing more effective vaccines than BCG remains a high priority in global TB control. The replacement of currently used BCG with different platforms might not be a practical goal because it is the most widely used vaccine in humans and has over 80% efficacy against severe forms of childhood TB, and because no other vaccine candidates (based on different platforms except BCG) are superior to BCG so far. Therefore, a vaccine that can work as an effective booster of BCG vaccine may be ideal because it will be more effective regardless of current vaccination strategy. In this study, our data clearly demonstrated that mice vaccinated with the recombinant plasmid pcD685A expressing the fusion protein of ESAT6 and Ag85A markedly reduced the bacterial load in the spleen and lung of vaccinated mice when compared with control group, but did not increase efficacy over BCG. More importantly, BCG prime followed by pcD685A DNA booster vaccination produced a greater protection as shown by reduced bacterial load and more pronounced antituberculous protective immune responses than BCG or pcD685A alone. The results of IFN-*γ* assay in a TST-positive population, cytokines assay of the lungs, postchallenge lung and spleen bacterial load, and pathological examination indicate that BCG prime plus pcD685A booster is an effective vaccine strategy against murine TB.

The antigen Ag85 complex was shared in by *M.tuberculosis*, BCG, and other mycobacteria, consisting of a family of three proteins (Ag85A, Ag85B, and Ag85C) with a MW range of 30–32000 daltons [12, 13]. These proteins possess mycolyl-transferase activity, which are responsible for the transfer of mycolic acids to a-a^'^-trehalose to form the cord factor and play an important role in the biogenesis of cell wall of mycobacteria [14]. The gene for ESAT-6 is absent in all BCG strains distributed worldwide, but is present in *M. tuberculosis* complex [15]. ESAT-6 is considered a dominant antigen for cell-mediated immunity [16] and is a major target for memory T cells in mice infected with *M*.* tuberculosis *[17]. Recently, different gene transferring systems such as modified vaccinia virus Ankara [18], alphavirus [19], vaccinia [20], or adenovirus [21, 22], BCG [23], and plasmid DNA [24–27] have been used to express Ag85A. The results of these studies showed that Ag85A can induce humoral and cell-mediated immunity and provide significant protection against TB in different animal models. Subunit vaccine based on antigen ESAT-6 also could enhance the efficacy of BCG [9], and the vaccine of the fusion protein of ESAT-6 with Ag85B which promotes strong and long-lived *M. tuberculosis*-specific T cell responses in naïve human volunteers has been evaluated in clinical study [28]. Recombinant BCG expressing ESAT-6 also conferred enhanced immunogenicity and protection against TB when compared with the parent BCG vaccine [29, 30]. Other vaccine platforms such as DNA vaccine [31–33], *Influenza* virus [34], and *Salmonella typhimurium* [35] also demonstrated that ESAT-6 is a potential immunodominant antigen for the development of TB vaccines. In addition, a chimeric protein of Ag85A and ESAT-6 with strong immunogenicity showed a treatment effect on MDR-TB in mice [36, 37]. In order to combine the immunological advantage of both antigens, we designed the fusion protein of Ag85A and ESAT-6 with a linker of 15 amino acid peptides, aimed to keep the naive configuration of these proteins. With the stimulation of purified r685A fusion protein, IFN-*γ* level in the peripheral blood from the TST-positive household contacts was much higher than that of TST negative group. Without the stimulation of r685A protein, IFN-*γ* levels in both groups are very low or none. These results indicate that the production of IFN-*γ* in the peripheral blood is r685A antigen specific and that r685A could stimulate the release of IFN-*γ* from sensitized T lymphocytes in the peripheral blood of household contacts. IFN-*γ* is an important indicator for Th1-type cellular immune response and is an essential mediator of the protective immune response to TB [38]. Therefore, we assume that the expression of r685A protein in vivo might be a promising stimulator of Th1-type cellular immune response against TB and might amplify the protective efficacy of BCG. In this study, pcD685A expressing r685A protein can protect mice against a primary *M. tuberculosis* infection with comparable pathological changes in lung with BCG, although bacterial load of lung and spleen in pcD685A vaccinated mice was higher than that of BCG. 

Previous studies have shown that repeat vaccination with BCG may be deleterious to protection against TB [39], and heterologous boost vaccines are likely to be used to enhance specific immunity primed by BCG [40] because BCG is not an effective booster vaccine itself. Several studies have demonstrated that recombinant modified vaccinia virus or adenovirus expressing Ag85A could enhance the waning BCG immunity and both have been under clinical evaluation [41, 42]. DNA vaccine expressing ESAT-6 [43] or the fusion protein of Ag85B with ESAT-6 also could amplify the protective immune responses of BCG prime [44, 45]. In our study, BCG prime and pcD685A booster showed the greatest protection against *M. tuberculosis *infection in mice. pcD685A could amplify antituberculous protective immune response in mice previously vaccinated with BCG, and markedly enhanced protection was correlated with increased IFN-*γ* level in the lungs of mice models prior to challenge. Although the gene encoding ESAT-6 is deleted in BCG, ESAT-6 may amplify overall anti-TB immunity in BCG immune animals by activating ESAT-6-specific T cells and play an important role in producing the booster effect of r685A protein.

## 5. Conclusion

In this study, our results clearly demonstrated the vaccination of C57BL/6 mice with DNA vaccine (pcD685A) expressing the fusion protein of Ag85A and ESAT-6 (r685A). pcD685A resulted in significant protection against challenge with virulent *M. tuberculosis* H37Rv when compared with the control group. Most importantly, BCG prime and pcD685A booster resulted in the most significant reduction in bacterial load of both spleen and lung when compared with BCG or pcD685A alone. Thus, our study indicates that pcD685A may be an efficient booster vaccine against TB with a strong ability to enhance prior BCG immunity.

## Supplementary Material

The cDNA squences of murine IFN-*γ*, IL-10 and GAPDH were obtained from GenBanK. Oligo primer analysis software was used to design PCR primers. F = sense; R = antisense.

## Figures and Tables

**Table 1:** Primers and thermal cycle parameters for cloning of *M. tuberculosis *antigens.

Protein Names	Primer names and sequences (5′-3′)	Cycle parameters
ESAT-6	ESAT6F: GGATCCATGACAGAGCAGCAGTG ESAT6R: GCTGCCGCCACCGCCGCTTCCGCCACCGCCGCTTCCACCGCCACCTGCGAACATCCCAGTGACGTTGCCTTC	95°C 5 min; 94°C 45 s, 60°C 45 s, 72°C 50 s, 30 cycles; 72°C 10 min
Ag85A	Ag85AF: GGTGGCGGTGGAAGCGGCGGTGGCGGAAGCGGCGGTGGCGGCAGCGCATTTTCCCGGCCGGGCTTG Ag85AR: GGAATTCTGTTCGGAGCTAGGCGCCCTGGG	95°C 5 min; 94°C 45 s, 60°C 45 s, 72°C 50 s, 30 cycles; 72°C 10 min
r685A	ESAT6F Ag85AR	95°C 5 min; 94°C 1 min, 60°C 1 min, 72°C 90 s, 30 cycles; 72°C 10 min
