# Supplementary material for: Immunogenicity and Protective Efficacy against Murine Tuberculosis of a Prime-Boost Regimen with BCG and a DNA Vaccine Expressing ESAT-6 and Ag85A Fusion Protein
Source: Clin Dev Immunol. 2011 Feb 27;2011:617892. doi: 10.1155/2011/617892 (PMC3065234; doi:10.1155/2011/617892)
Supplement: Supplementary file 1 — The cDNA squences of murine IFN-γ, IL-10 and GAPDH were obtained from GenBanK. Oligo primer analysis software was used to design PCR primers. F = sense; R = antisense. [file 617892.f1.pdf]

Supplementary Table 1. Primer sequences and cycle parameters for murine cytokines by qRT-PCR analysis

| | | Primer sequences（5'-3'） | Cycle parameters |
|---|---|---|---|
| GAPDH | F | GACCAGGTTGTCTCCTGCGACTTC | 95 °C 3min; 40 cylces of 95°C 15s, 60 °C 20s, 72 °C 20s; |
| | R | GGTGGGTGGTCCAGGGTTTCTTAC | |
| IFN-γ | F | GGCTGTCCCTGAAAGAAAGC | |
| | R | GAGCGAGTTATTTGTCATTCGG | |
| IL-10 | F | GGTTGCCAAGCCTTATCGGA | |
| | R | ACCTGCTCCACTGCCTTGCT | |
